# Factors driving the implementation of the ‘Local New Year’ policy to prevent COVID-19 in China

**DOI:** 10.1057/s41599-023-01765-0

**Published:** 2023-05-26

**Authors:** Bifeng Zhu, Manqi Ding, Xingwei Xiang, Chaoyang Sun, Xiaoqian Tian, Junfeng Yin

**Affiliations:** 1 College of Urban Construction, Zhejiang Shuren University, Hangzhou, China; 2 College of Architecture, Zhejiang University of Technology Zhijiang College, Shaoxing, China; 3 School of Architecture and Urban Planning, Huazhong University of Science and Technology, Wuhan, China; 4 Zhejiang Yongze Architectural Design Co., Ltd, Hangzhou, China

**Keywords:** Theatre and performance studies, Social policy, Psychology

## Abstract

This study examines the contradiction caused by the ‘local new year’ policy, that is, the conflict between the pandemic prevention policies and people’s emotional demands during the Spring Festival, based on the normalisation of pandemic prevention and control. It focuses on the scientific logical relationship with the contradiction that people voluntarily support ‘local new year’, to explore the primary driving factors of their willingness. By evaluating the migrant workers in large cities, the primary influencing factors were screened, and the primary dynamic factors and their relationship were obtained using the Logit logical selection model and maximum-likelihood estimation. The study identified, ‘whether social and entertainment activities are planned in migrant cities’, as the primary driving factor, followed by ‘whether there are relatives (elderly /children) at home’, and ‘contracting the infection during travel’. In view of this conclusion, this study further proposes corresponding policy suggestions: Relevant measures should be adopted according to different regions and the current situation of the pandemic in combination with the characteristics of the episodic and local nature of the pandemic. ‘Local new year’ is encouraged from the perspective of enriching people’s emotional needs for spiritual entertainment and care. This study provides a new perspective and theoretical basis for the research and formulation of policies related to the normalisation of pandemic prevention and control in China and worldwide, and has a certain practical reference value.

## Introduction

Spring Festival Transportation (SFT), (transportation of passengers and goods during the Spring Festival), is a large-scale transportation phenomenon around the Spring Festival of the Chinese lunar calendar. The SFT lasts for ~40 days, from the 15th day of the twelfth lunar month to the 25th day of the first month of the new year. It involves more than 2 billion people, accounting for one-third of the world’s total population (Lai and Pan, 2019). China’s SFT is known as the largest periodic human migration in human history (Duan et al., 2009).

Since China’s reform of social production relations and opening up in 1980, the productivity has improved, which has led to the rapid development of cities and towns and the flow of population from rural areas to cities (Bettencourt et al., 2007). With the relaxation of restrictions on personnel mobility by the Chinese government, people increasingly opt to leave their hometown to work and study in economically developed cities and towns or southeast coastal areas (Kenneth, 2018). SFT is the embodiment of the life of Chinese folk culture (Fan, 2019). It is the cherished wish of every Chinese living thousands of miles away from home, to return home on the day of the Spring Festival. Therefore, the SFT not only represents the special period of Chinese passenger transportation but also a unique cultural phenomenon in China.

According to the statistics released by China’s Ministry of Transport, the passenger traffic volume of SFT reached 2.91 billion in 2016, 2.981 billion in 2017, 2.98 billion in 2018 and 2.99 billion in 2019. Data on SFT passengers for four consecutive years reflect that the traffic volume maintained a stable high flow and increased slightly. However, the passenger traffic volume of SFT was only 1.476 billion, in 2020, a year-on-year decrease of 50.3% compared with 2019 (Xiang et al., 2021). Among them, 1.143 billion passengers travelled on the 15th day before the festival, a year-on-year increase of 2% over 2019. On the 25th day after the Spring Festival, 333 million passengers travelled, a year-on-year decrease of 82% compared with 2019 (Dai et al., 2020). The primary reason for this trend was the outbreak of the COVID-19 pandemic in some cities and regions in China since the beginning of February 2020. To prevent the continuous spread of the pandemic, most parts of China had postponed working hours after the festival to reduce the spread of the virus caused by personnel mobility. Simultaneously, the government had imposed traffic control on the return trip of the SFT. Therefore, compared with previous years, there was no peak of returning to work after the Spring Festival holiday in 2020. Subsequently, the passenger traffic volume of SFT in 2021 was only 870 million, a decrease of 70.9% over the same period in 2019 and 40.8% over the same period in 2020 (Yang, 2021). This indicates that the SFT was significantly affected by the pandemic, and the transportation volume and scale of the SFT remain significantly reduced considering the normalisation of prevention and control of the pandemic after 2020.

China’s major initiative to control the outbreak of COVID-19 during the Spring Festival is the ‘Local new year’ (staying in the city where one works and not going back to one’s hometown for the Spring Festival) policy. The policy had significant effect when the first outbreak of the pandemic occurred in China in February 2020 (Sun and Gan, 2021). During the Spring Festival of 2020, people actively cooperated with the government to achieve ‘local isolation’, so that the pandemic could be controlled within a short span, ensuring an unhindered prevention and control of the pandemic in China (Adekola et al., 2020). In terms of the COVID-19 control mechanism, the government adopted measures such as early and strict blockade, active case monitoring, rapid diagnosis and effective contact tracking, so that China would be recognised by the international community in the management of current and future public health emergencies (Lin et al., 2021). In particular, after the COVID-19 outbreak in Wuhan, the Chinese government played the role of big data technology in the early warning and monitoring of COVID-19, and actively and dynamically adjusted the pandemic control policy to prevent the spread of the virus (Wu et al., 2020).

However, despite the normalisation of prevention and control of the pandemic, the ‘local new year’ was proposed again, on the eve of the Spring Festival in 2022. However, doubts such as, ‘the managers of areas that advocate “local new year” are irresponsible’ were common. Undoubtedly, the advocacy of ‘local new year’ caused discomfort to some people during this special period. The policy of ‘local new year’ requires people to stay in their city of work during the Spring Festival and not visit their hometowns, which conflicts with people’s emotional need to visit their hometown and reunite with their families. Therefore, two opposing voices were identified. Those in favour believed that the proposal of ‘local new year’ followed the experience of previous years that had achieved remarkable results in preventing the spread of the virus. The implementation of ‘local new year’ could ensure the pandemic prevention to the fullest extent, and should be vigorously advocated (Xu et al., 2021; Wang et al., 2020; Abel Brodeur et al., 2021, Clark et al., 2021). However, opponents believed that the serious pandemic stage had passed and people were no longer afraid of the pandemic, therefore, the ‘local new year’ during the Spring Festival was an excessive pandemic prevention behaviour. In addition, the continuous yearning to visit their hometown far exceeded their concern about the pandemic. They believed that it was unnecessary to implement the ‘local new year’ at all (Chu et al., 2020; Liu and Song, 2020; Huang and Xu, 2020). The primary reason for the contradiction was the people’s desire to visit their hometown during the Spring Festival, considering the gradual normalisation of pandemic prevention and control in China and the approaching Spring Festival holiday in 2022. However, once again the government advocated ‘local new year’, which was difficult to obtain people’s general psychological recognition.

Previous studies reveal that population mobility accelerates the spread of the pandemic. For everyone standard deviation increase in population mobility, the number of confirmed cases will increase by 12.8% (Li and He, 2020). Reducing personnel mobility can effectively reduce the infection rate (Amaku et al., 2021). Therefore, from the perspective of effective pandemic prevention, ‘local new year’ is considered to be one of the most effective and direct means of pandemic prevention during the Spring Festival, which is worthy of promotion and implementation (Ammar et al., 2020).

In addition to forming relevant policies, the pandemic transformed human behaviours. Alireza Dianat et al. observed the changes in the frequency and pattern of the five most repetitive tasks (work, shopping, cooking, eating, and visiting relatives and friends) in people’s daily arrangements during the pandemic (Alireza Dianat et al., 2022). Other related studies revealed that people increasingly liked to cook at home, were reluctant to shop in person, and preferred to work from home (Rezwana Rafiq et al., 2022; Brynjolfsson et al., 2020). People generally reduced travelling and visiting friends (Alireza Dianat et al., 2022). The aforementioned behavioural changes reduced the opportunities for people to contact each other, thus reducing the spread of the pandemic. In terms of mode of commuting, Tonmoy Paul and others proposed that during the outbreak of the pandemic, people preferred walking, using private cars and bicycles, and the number of people who opted for public transportation was significantly reduced (Tonmoy Paul et al., 2022). In terms of travel frequency, Ruth Steiner et al. concluded that the number of people staying at home during the pandemic increased significantly (Ruth Steiner and Chen, 2022). The above studies were about the impact of COVID-19 on human behaviour, and primarily focused on the level of the phenomenon. However, the logic behind people’s behavioural changes regarding these phenomena were rarely discussed. The specific factors that affect people’s behavioural choices, lack an in-depth analysis and research, which is not conducive to forming the basis for effective and easily accepted policy formulation.

China’s pandemic prevention policy proposal is based on the requirement to limit the spread of the virus and conforms to the characteristics of people’s behavioural changes under the pandemic. However, during the Spring Festival, which is culturally significant to the Chinese, people are required to follow the ‘local new year’ policy by means of administrative orders. This inevitably triggers social conflicts as it is difficult for the masses to accept the order. An effective mechanism for pandemic prevention involves formulating relevant policies to guide people to actively respond to them. Moreover, the humanised measures are implemented to assist the implementation of the relevant provisions of the policy. It can prevent and control the pandemic situation to the maximum extent, considering the emotional needs of people (Jie Peng, 2021). Therefore, we believe that the focus of the contradiction is not whether to implement ‘local new year’, but the lack of care for people’s emotional needs and the operability of policy implementation. To promote the policy of ‘local new year’, it is necessary to understand the scientific and logical relationship with the contradiction that people voluntarily select ‘local new year’ and determine its primary driving factors.

This study discusses the social focus of China’s ‘local new year’ policy under the normalisation of pandemic prevention and control. It attempts to determine the primary driving factors responsible for the implementation of the ‘local new year’ by evaluating public opinion, expects to formulate relevant specific policies and measures based on the identified driving factors, and construct a detailed implementation plan for the ‘local new year’ policy. This study is useful as it attempts to maximise the implementation of pandemic prevention policies while considering the needs of people’s spiritual culture and social activities. It provides a new perspective and theoretical basis for the research and formulation, implementation, and practice of policies related to global pandemic normalisation prevention and control.

The innovations of this study:

(1) Innovative research from the perspective of determining influencing factors: This study examines Chinese pandemic prevention policy of ‘local new year’. The focus of the study is not to discuss the formation and advantages of the policy, but to focus on the determinants responsible for behavioural change brought about by the pandemic, so as to analyse the ‘social contradictions’ caused by the policy. The scientific and logical relationship between pandemic prevention policy-making and behavioural decision-making is clarified. For large-scale traffic phenomena such as the Spring Festival, during the period of COVID-19 or any other pandemic, policies are formulated from the perspective of people’s behaviour determinants to focus on people’s emotional needs, so as to achieve the efficient implementation of policies.

(2) Innovation in the application of research methods: Based on the public survey data, the study uses Cp method to screen out the major factors affecting people’s ‘local new year’ behaviour. On this basis, the relationship between the major factors is studied by constructing the Logit model of ‘local new year’ behaviour choice. The combined application of Cp Method and Logit model analysis ensures the accuracy of the research object and the scientificity of the research process.

## Prevention and control policies applied in Chinese cities during the spring festival

In early 2020, COVID-19 broke out in Wuhan, China. The Chinese government launched a major response mechanism for public health emergencies, which controlled the flow of passengers during the Spring Festival. Cities and districts introduced policies to prevent and control the pandemic situation. To effectively control the spread of the pandemic, some cities and regions closed up and monitored (Almudéver Campo and Camaño Puig, 2020), proposed the ‘local new year’, and called on everyone not to return home for the Spring Festival (Chi, 2020).

Although the pandemic situation in China remained stable in 2021, the coexistence of local distribution and large-scale aggregation, which was in the state of normalised prevention and control, continued. It coincided with China’s SFT in 2022. According to statistics, as of 22 December 2021, at least 55 regions (accounting for 8% of the total) in China had issued policies advocating ‘local new year’ on New Year’s Day and Spring Festival in 2022 (NDRC, 2021). However, government departments in different regions indicate certain differences in the implementation of the specific policies of ‘local new year’ (see Table 1).

**Table 1:** Specific policies of each province (region) on ‘local new year’.

Province	City (region)	Specific policies on ‘local new year’
Ningxia	Yinchuan	The government discouraged people from going on business trips or travelling unnecessarily, and encouraged people to spend the new year’s holiday locally.
Guangdong	Zhongshan	Enterprises encouraged employees to celebrate the Spring Festival locally, and entrepreneurs were advised to take the lead in organising an enjoyable ‘local new year’ for employees, and form a consensus that ‘it is not necessary to leave Guangdong’.
Guangxi	Pingxiang	Issued the ‘ten uniform’ notice, emphasising that as the end of the New Year was approaching, stores, shops should reduce personnel flow and advocate assistants to celebrate ‘local new year’ or online New Year.
Hebei	Zhangjiakou (Economic development zone)	All cadres and staff of the Party and government organisations, public institutions, and state-owned enterprises in the region were instructed spend the Spring Festival in local area. Further, they were instructed not to travel to high-risk areas or leave the local area. Functional departments of the region were instructed to ensure the guarantee of water, electricity, gas, communication, and heating to improve the supply capacity of daily necessities, pandemic prevention materials and drugs.
	Beijing	The government will strengthen market monitoring of daily necessities to ensure price stability and adequate supply, expand the supply of public culture, enrich people’s festive cultural life, and improve the quality of life services and humanistic care for students, teachers and migrant workers in Beijing.
Zhejiang	Ningbo	The enterprise will grant special subsidies to employees of non-Ningbo enterprises. The standard subsidy is 100 Yuan per person per day, and the maximum subsidy per person will be 500 Yuan.
	Shanghai	Foreign cadastral personnel will receive a one-time subsidy of 700 Yuan for ‘as gift for stay in Shanghai and overcoming living difficulties’.
Fujian	Xiamen	During the Spring Festival, each person who stays in Xiamen without household registration will be provided 50 Yuan in cash, and those who work in service industry will be provided a subsidy of 50 Yuan per day.
Jiangsu	Suzhou (Xiangcheng District)	The enterprise will provide 500 Yuan subsidy to each foreign employee who stays in Xiangcheng. During the Spring Festival, enterprises should rent dormitories, blue-collar apartments and talent apartments for foreign employees to live in, with half a month’s rent reduced.

To sum up, according to the degree of concern for people’s feelings, China’s ‘local new year’ policies in various regions were divided into three types:

(1) The policy of ‘local new year’ was enforced, and the government blindly required people to comply with pandemic prevention regulations, lacking relevant supporting emotional care measures.

(2) From the perspective of morality, people were required to implement the policy of ‘local new year’. Simultaneously, material supply was ensured and mass cultural activities were conducted during the Spring Festival.

(3) On the premise of meeting people’s actual life and spiritual needs, the relevant policies of ‘local new year’ were implemented flexibly according to specific conditions. Simultaneously, a series of measures were adopted to assist in the implementation of the policy, such as monetary subsidies and compensation for those who stayed in the their city of work.

## Methodology

### Data and samples

The data were obtained on two different occasions: the first survey was conducted to obtain the interviewees’ opinion of the possible factors that affect the ‘local new year’, so as to formulate a unified influencing factor table (see Table 2) for the second survey.

**Table 2:** Influencing factors.

Number	Influencing factors
1	Whether you are satisfied with your income this year?
2	Do you have any relatives (elderly /children) in your hometown?
3	Whether the government called for ‘local new year’?
4	Can you buy the ticket?
5	Whether subsidies or consumer welfare have been provided for the ‘local new year’?
6	Whether the possibility to contract COVID-19 during travel will affect the decision to go home for the festival?
7	Will you change your job in the next year?
8	Do you have the traditional sense of visiting your hometown for the Spring Festival?
9	Whether related activities have been arranged in hometown (such as weddings, parties)?
10	Whether activities have been arranged in the city of work?
11	Is the transportation convenient for travelling to your hometown?

The second survey aimed to obtain the characteristic information of the interviewees, primarily including, (a) their basic information, and (b) the characteristic information of the influencing factors. We classified and assigned the characteristic information in the second questionnaire. The results are presented in Table 3.

**Table 3:** Classification and assignment of variables.

Name	Type	Variable	Options	Assignment
_COL1	Discrete	Age	20–30	1
			30–40	2
			40–50	3
			50–60	4
_COL2	Discrete	Occupation	Service worker	1
			Company employees	2
			Professional and technical personnel	3
			Official	4
			Migrant workers/construction workers	5
			Other	6
_COL3	Discrete	Income range (YUAN/month)	under 2000	1
			2000–5000	2
			5000–10000	3
			Over 10000	4
_COL4	Discrete	Hometown type	City	1
			Rural	2
			County/town	3
_COL5	Discrete	The distance of the hometown	Inter-province	1
			Neighbouring province	2
			Intra-province	3
_COL6	Binary	Gender	Male Female	1 0
_COL7	Binary	Is your family around?	Yes No	1 0
_COL8	Binary	Do you have children?	Yes No	1 0
_COL9	Binary	Are you satisfied with your income this year?	Yes No	1 0
_COL10	Binary	Do you have any family members(elderly /children) in hometown?	Yes No	1 0
_COL11	Binary	Whether the government called for ‘local new year’?	Yes No	1 0
_COL12	Binary	Can you buy the ticket?	Yes No	1 0
_COL13	Binary	Whether subsidies or consumption welfare have been provided for the ‘local new year’?	Yes No	1 0
_COL14	Binary	Is it possible to contract COVID-19 while travelling?	Yes No	1 0
_COL15	Binary	Will you change your job in the next year?	Yes No	1 0
_COL16	Binary variable	The sense of traditional reunion	Yes No	1 0
_COL17	Binary	Whether related activities have been arranged at home (such as weddings, parties)?	Yes No	1 0
_COL18	Binary	Whether activities have been arranged in the city of work?	Yes No	1 0
_COL19	Binary	Is it convenient to travel home?	Yes No	1 0

The data collection period was December 2021, on the eve of China’s 2022 SFT, so as to accurately collect information about people’s willingness to travel home during the Spring Festival. The questionnaire was distributed online, and covered East, North, Southwest, and Southeast China. Total 100 questionnaires were distributed for the first time and 600 for the second time. Total 584 valid questionnaires were collected in the second survey, and the effective rate is about 97.3%. The basic information of interviewees is presented in Table 4.

**Table 4:** Basic information of interviewees.

Content	Composition proportion
Gender	Male 52.7%, Female 47.3%
Age	20–30 years old 29.17%; 30–40 years old 32.5%; 40–50years old 36.67%; 50–60 years old 1.67%
Occupation	Migrant workers/construction workers: 33.33%; official: 10%; company employees: 10%; professional and technical personnel: 5%; service workers: 25%; other: 16.67%
Income (YUAN/month)	Under 2000: 8.33%; 2000–5000: 49.17%; 5000–10,000: 32.5%; Over 10,000: 1.67%
Children	Have children: 75%; Do not have children: 25%
Family around	Yes: 71.67%; No: 28.33%
Hometown	Rural: 78.33%; County town: 10%; City: 11.67%
Distance	Inter-province: 67.5%; Neighbouring province: 7.5%; Intra-province: 25%
Whether to go home for the Spring Festival	Yes: 93.33%; No: 6.67%

### Research method

#### Cp method

Cp method is proposed by Mallows’ Cp. Mallows used Cp value to evaluate the optimal combination of a linear regression model based on the assumption of ordinary least square method (Fadaee et al., 2020). It is based on the original regression model, and the Cp statistical value of a sub-regression model can be defined as:

$${\mathrm{Cp}} = \frac{{{\mathrm{SSE}}_{{\mathrm{reduced}}}}}{{{\mathrm{MSE}}_{{\mathrm{Full}}}}} - n + 2p$$

‘MSE’ is the mean square error; ‘SSE’ is the sum of square error. Assuming that there are *K* sets of independent variables in total, *p* sets of independent variables are selected from *K* sets to establish a sub model, and ‘*n*’ is the sample size, then the formula substituted is:

$${\mathrm{Cp}} = \frac{{{\mathrm{SSE}}_{{\mathrm{reduced}}}}}{{{\mathrm{MSE}}_{{\mathrm{Full}}}}} - n + 2p = \frac{{{\mathrm{SSE}}_p}}{{{\mathrm{MSE}}_K}} - n + 2p$$

${\mathrm{SSE}}_p = {\textstyle{1 \over {{{{\mathrm{n}}}} - {{{\mathrm{p}}}}}}}{\mathrm{MSE}}_p$, ‘MSE_p_’ is the mean square error in the sub-regression model. Then the formula substituted is:

$${\mathrm{Cp}} = \frac{{{\mathrm{SSE}}_p}}{{{\mathrm{MSE}}_K}} - n + 2p = \left( {n - p} \right)\left[ {\frac{{{\mathrm{MSE}}_p}}{{{\mathrm{MSE}}_K}} - 1} \right] + p$$

When Cp ≤ *p*, the screening can be stopped and the self-variable quantum set is considered to be the best combination. It not only simplifies the model by using a small number of independent variable combinations, but also keeps the mean square error of the model unchanged or reduced, so as to effectively prevent the occurrence of over fitting, resulting in the decline of the prediction ability of the model (Srivastava et al., 2014). The selection of self-variable quantum sets by Cp method can effectively control the number of parameters, which significantly reduces the error rate in the process of model calculation (Popescu and Gheorghiu, 2021).

#### Logit model and maximum-likelihood estimation

Logit is a discrete selection model, which belongs to the category of multivariate analysis. Marschark (1960) proved the consistency between Logit model and maximum utility theory. Marley (1965) studied the relationship between the form of the model and the distribution of the utility uncertainty, proving that the extreme value distribution can deduce the Logit form of the model (Baidu Baike, 2021). Moreover, Logit model has been proved by McFadden that it must obey the extreme value distribution, so that it verifies the rationality for solving the problem of logical selection. Logit model has the dominant feature of probability expression. When the model selection set does not change, but the level of each variable changes, the selection probability of each selection branch in the new environment can be easily solved (Vani Borooah, 2012). In the process of Logit calculation, an important and universal method for calculating estimators is used, that is, maximum-likelihood estimation. It is representative of a class of completely statistical phylogenetic tree reconstruction methods. According to the probability model, it seeks a phylogenetic tree that can generate observation data with a high probability (Matthew et al., 2020).

In this study, ‘local new year’ is set as the dependent variable (binary variable), and the basic form of Logit model is used to model the probability *P* of something.

**Logit** (***P***_**i**_) = ***β***_**0**_ + ***β***_**1**_***X***

*β*_0_ and *β*_1_ are the coefficients of functional regression, and *X* is the independent variable. *β* and *X* are regarded as vector forms, then:

**Logit** (***P***_**i**_) **=** **ln(p**_**i**_**/(1−p**_**i**_)) = ***β***_**0**_ + ***β***_**1**_***X***_**1,i**_ + ***β***_**2**_***X***_**2,i**_ + **···+*β***_**n**_***X***_***n*,i**_

The occurrence possibility of dependent variable is *P*_1_, that is: *P*_1_ = *P* (Possibility = 1); Correspondingly, the possibility of non-occurrence is *P*_0_, that is, *P*_0_ = *P* (Possibility = 0). *P*_1_ + *P*_0_ = 1.

Establishing Logit equation:

Logit = log(Odds) **=** ln(*P*_1_/*P*_0_) = *β*_0_ + *β*_X_

To avoid the problem of complete separation in data structure, regression equation is established:

Logit = log(Odds) **=** ln(*P*_1_/*P*_0_) = *β*_0_ + *β*_1_·*X*_1_ + *β*_2_·*X*_2_ + *β*_3_·*X*_3_ + ……

### Research framework and steps

**Fig. 1:**
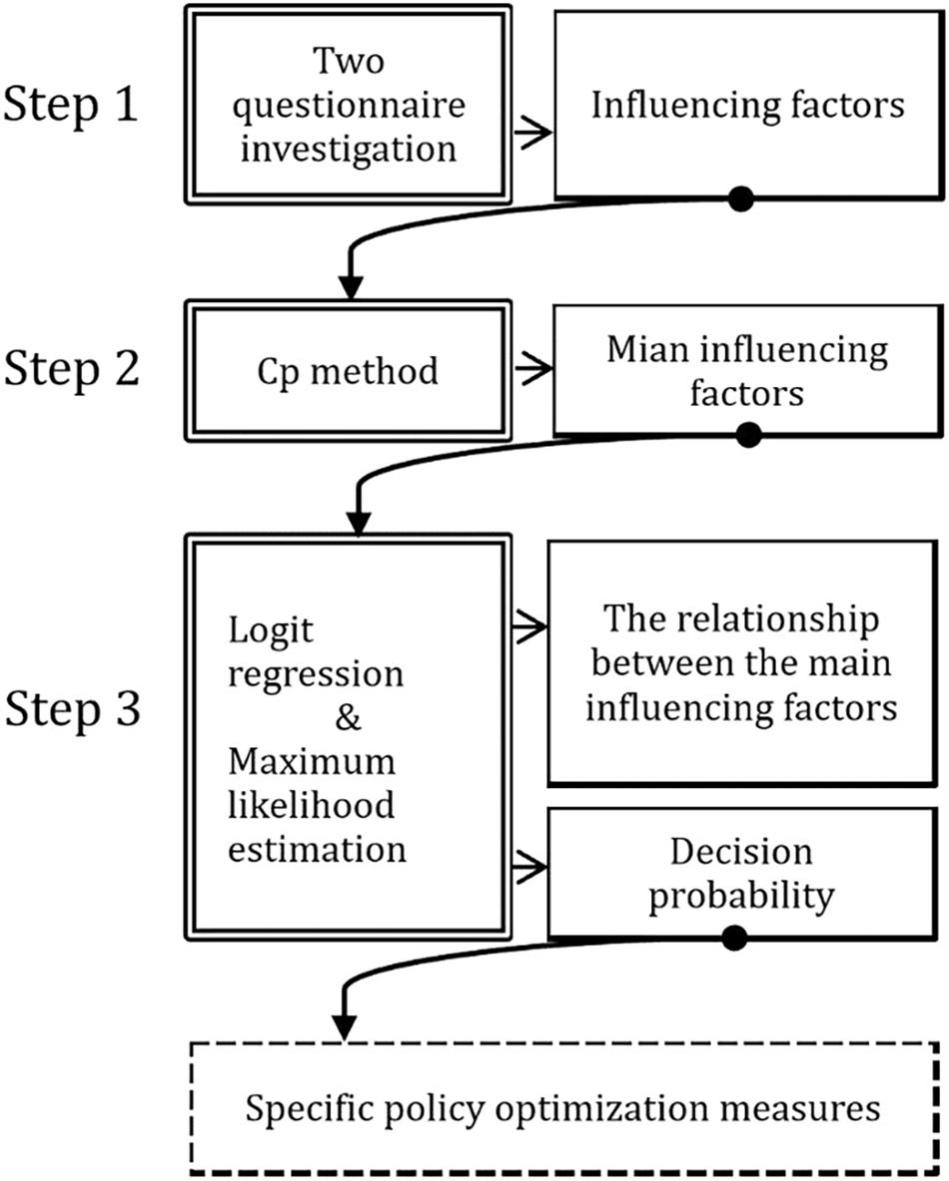
Research framework. This study is divided into three steps to obtain the influencing factors, main influencing factors, and the relationships between the main factors to achieve the optimization of specific policies.

This study is divided into three steps (see Fig. 1).

**Step 1: Obtaining possible influencing factors**

Using a questionnaire, a random survey was conducted on migrant workers in large cities to obtain their opinion about the factors they believe affect the ‘local new year’. Based on the recovery of the questionnaire, the validity of the answer to the questionnaire and the mentioned rate of factors (more than 10%), these influencing factors were preliminarily screened to determine the possible factor table.

**Step 2: Screening out the primary influencing factors**

Based on the possible factors table developed in Step 1, the second survey was conducted. The data collected from the survey were screened using the Cp method to obtain the primary influencing factors.

**Step 3: Analysing the relationship between the primary factors**

Considering the primary influencing factors obtained by the Cp method, the relationship between the primary influencing factors and the selection probability of whether to implement the ‘local new year’ were discussed by using logical selection model and maximum-likelihood estimation method, so as to further clarify the direction of policy-making in the future.

## Discussion on primary influencing factors

The calculation table was obtained through the simulation calculation of Cp method. The smaller Cp value indicates the better fitting of the model. Owing to the possibility of too many variable combinations, the calculation results reveal the top five combinations with the best fitting degree (see Table 5).

**Table 5:** Calculation results of the Cp method.

Number in model	Cp	*R* square	Variables in the model
4	−3.1388	0.1834	_COL10 _COL12 _COL14 _COL18
3	−2.9445	0.1666	_COL10 _COL14 _COL18
4	−2.2445	0.1766	_COL7 _COL10 _COL14 _COL18
5	−1.9871	0.1899	_COL10 _COL12 _COL13 _COL14 _COL18
5	−1.9689	0.1897	_COL7 _COL10 _COL12 _COL14 _COL18

Among the five groups of data in the above table, the first group has the smallest Cp value and its fitting effect is the best. The results reveal that the significant variables are: _COL10 _COL12 _COL14 _COL18. They are the four primary factors that determine whether people support ‘local new year’: (1) Whether there are relatives (elderly /children) in their hometown? (2) Can buy the ticket? (3) Risk of contracting COVID-19 while travelling; (4) Whether any activities were arranged in the city of work?

According to the four primary factors selected, travelling home to visit the elderly and reunite with children is the most significant driving force for people to return home during the Spring Festival. China’s public transport bears huge pressure every year owing to the high passenger flow momentum of the SFT (Li et al., 2016). ‘One ticket is difficult to obtain’ has become a common complaint that has plagued Chinese people’s travel during the Spring Festival for several years (Zhou, 2012). Particularly, during the peak period of passenger travel around the Spring Festival, whether people can buy a ticket for their hometown has become a realistic factor for people to travel home for the festival. People’s anxiety about contracting COVID-19 while travelling is an important psychological factor in deciding whether to visit home for the Spring Festival. Among the four primary factors, the results for the factor, ‘Whether activities were arranged in their city of work’ were different from the expected results. It revealed that people were willing to celebrate the ‘local new year’, if interesting arrangements were organised for the Spring Festival. For Chinese people who remain busy over the year, the seven-day holiday during the Spring Festival is a rare holiday (Ahn, 2021). The extensive holiday arrangements make people feel relaxed. For individuals, if more activities can be arranged in their city of work, people will opt to stay in that city for the Spring Festival, otherwise they may become bored and opt to leave or travel back to their hometown. Therefore, ‘whether activities were arranged in their city of work’ became one of the primary influencing factors.

**Fig. 2:**
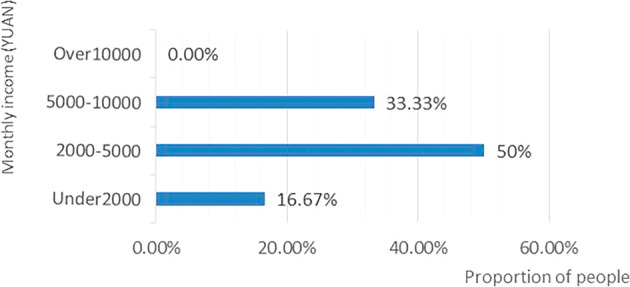
Distribution of people in each income segment who choose the factor of “subsidies or consumption welfare”. Most respondents who choose the factor of "subsidies or consumption welfare" have a monthly income between 2000-10000 yuan.

As currency is generally regarded as the most direct means of compensation (Isdale and Walpole, 2020), the governments identify cash and other material subsidies as the most attractive factor for migrant workers in formulating pandemic prevention policies (Liu, 2020). To retain several workers to celebrate ‘local new year’ without travelling, several regional governments used cash subsidies or consumption vouchers (Sutherland et al., 2013). However, statistical data reveals that only 5% of people opted for cash subsidies and consumption vouchers as the primary factor for supporting ‘local new year’. Among them, 0.8% of people with a monthly income of less than 2000 yuan, 2.5% of people with a monthly income of 2000–5000 yuan, and 1.7% of people with a monthly income of 5000–10000 yuan opted for this factor (see Fig. 2). It is clear from the correlation between income and material subsidy demand that no higher proportion of low-income people opted for this factor. Therefore, it indicates that material subsidies such as cash subsidies and consumption vouchers are insufficient to attract people to support ‘local new year’. The primary reason why people opt for ‘local new year’ is not the generally recognised cash vouchers and consumption subsidies (Lin et al., 2020), but the extensive festival arrangements, indicating that people’s needs are more spiritual than material.

## Discussion on the relationship between the primary factors

The simulation calculation of SAS (Statistical Analysis System) 9.4 was used to detect whether each variable in the model is significant. _COL10, _COL14, _CoL18 are significant because their Chi Square values are less than 0.05, that is, the confidence values of variables are more than 95%. The Chi Square value of _COL12 is 0.3736, that is, confidence value is 62.624%, therefore, the variable is not significant (see Table 6). Therefore, it can be determined that the independent variables of this study are the factors, ‘Whether there are relatives (elderly /children)’, ‘Risk of contracting COVID-19 during travel’, and ‘Whether activities were arranged in the city of work’.

**Table 6:** Analysis of maximum-likelihood estimates.

Factor	DF	Estimate	Standard error	Wald Chi Square	Pr > ChiSq
Intercept	1	1.9826	0.6261	10.0279	0.0015
_COL10	1	2.0164	0.9028	4.9881	0.0255
_COL12	1	1.1672	1.3117	0.7918	0.3736
_COL14	1	−2.1320	0.9530	5.0051	0.0253
_COL18	1	−4.5826	1.7591	6.7866	0.0092

The parameter estimates of the three factors determined were calculated, and the results are presented in Table 7.

**Table 7:** Odds ratio estimates.

Factor	Point estimation	95% Wald confidence limits	
_COL10	7.511	1.280	44.079
_COL14	0.119	0.018	0.768
_COL18	0.010	<0.001	0.321

The results reveal that for ‘whether there are relatives (elderly /children) at home’, the probability to visit home is 7.511 times higher than those without relatives. When other variables remain unchanged, people who do not care ‘whether they will contract COVID-19 during travel’ are 8.4 (1/0.119) times more likely to go home than those who are afraid of contracting COVID-19 during travel. When other quantities remain unchanged, people who have ‘no activity arrangement in city of work’ are 100 (1/0.01) times more likely to go home than those who have arrangement.

The above data reveals that ‘whether there are relatives (elderly /children) at home’, ‘the risk of contracting COVID-19 during travel’, and ‘whether activities were arranged in city of work’ are the three primary direct factors that lead people to support ‘local new year’. When other factors remain unchanged, the probability of returning home is 100 times higher for those who have ‘no activity arrangement in city of work’ than those who have arrangement, which is much higher than 8.4 times the impact of the ‘risk of contracting COVID-19 during travel’, and 7.511 times the impact of ‘whether they have relatives (elderly/children) at home’. Therefore, the ‘arrangement of activities in city of work’ is the most important and direct determinant of the three influencing factors, indicating that this factor is the primary driving factor for the adjustment and formulation of the ‘local new year’ policy. In addition, when other factors remain unchanged, people who do not care ‘whether they will contract COVID-19 during travel’ are 8.4 times more likely to go home than those who are afraid of contracting the infection, which is slightly higher than 7.511 times the influence of ‘whether there are relatives (elderly/children) at home’ on the probability of going home. Data reveals that the difference between the two is minimal. Thus, the importance of the two is similar, and they are the secondary influencing factors for people to support ‘local new year’. It indicates that the two factors simultaneously play a secondary driving role in the adjustment and formulation of the ‘local new year’ policy.

## Suggestions on policy formulation and implementation

Based on the primary and secondary influencing factors obtained from the study, the following adjustments are suggested for the formulation and implementation of China’s ‘local new year’ policy.

### Policy formulation

(1) For areas with serious pandemic development, such as Xi’an, Tianjin, Dongguan, and other places (considering the Spring Festival in 2022), to prevent the possibility of further spread of the pandemic in these areas, the ‘local new year’ policy should be strictly implemented. Emphasis should be laid on people’s responsibility and obligation to abide by the pandemic prevention regulations, and the government should strengthen the guarantee of water, electricity, gas, heating and so on, so as to ensure the supply capacity of daily necessities, pandemic prevention materials and drugs (Yu and Yu, 2021). The distribution of cash subsidies can be reduced or cancelled, and the supply of public cultural and recreational activities can be increased to enrich citizens’ experience of the Festival on the premise of reducing aggregation and self-protection. In addition, local government can help the elderly and children in their hometowns by solving their problems, and providing ‘online new year greetings’, services, and support (Wang and Mao, 2021). For special groups and families, the risk of virus transmission can be controlled to the fullest extent by means of point-to-point isolation and flow such as using chartered cars.

(2) For areas where the pandemic is in the development stage and there are risks of subsequent transmission, such as Shaoxing, Ningbo, Shanghai, Jiangsu and other places (considering the Spring Festival in 2022), people should be encouraged to support ‘local new year’ and the market monitoring of daily necessities should be strengthened to ensure stable prices and sufficient supply (Tomal and Helbich, 2022; Zhu et al., 2021). The government can ensure the improvement of the material supply and quality of cultural life of non-locals during the Spring Festival, cancel the payment of cash subsidies, focus on the fulfilment of the spiritual needs of non-locals and provide humanistic care.

(3) For areas where the pandemic situation is under control and the pandemic situation is in the final stage or there are no new cases for several consecutive days, such as Harbin, Qiqihar, Yunnan, Inner Mongolia and other places (considering the Spring Festival in 2022), policies should be formulated according to the pandemic situation of the destination. For low-risk destinations, it is not recommended to propose the policy of ‘local new year’, and people should have the option to freely decide whether to travel home to reunite with their relatives. However, for medium and high-risk areas, it is recommended to implement ‘local new year’ or travel to other low-risk areas for festival, to avoid returning to hometowns (Wibbens et al., 2020).

### Implementation suggestions

Of the three factors, comparing with the other two factors, ‘the possibility of contracting COVID-19 infection during travel’ is significantly influenced by the objective situation of the pandemic development, and is not subject to subjective policy control. Therefore, policy implementation should focus on the other two factors, that is, the local government should focus on the spiritual needs of the groups supporting ‘local new year’ and the living needs of the elderly and children in hometowns (Mbazzi et al., 2020).

(1) **Focusing on the spiritual needs of groups favouring ‘local new year’**. Local governments and enterprises should first ensure the supply of basic materials for non-locals during the Spring Festival, and thereafter, focus on their spiritual care. During the Spring Festival, government representatives should visit and comfort those supporting the ‘local new year’, to ease their discomfort of not being permitted to visit their hometowns and reuniting with their relatives. For example, street communities and unions can prepare ‘New Year’s Eve dinner’ and organise non-locals to make dumplings, so that they can feel the festive atmosphere of the traditional Spring Festival. Their homesickness is alleviated by way of ‘online reunion’ (Hu and Jiang, 2021). Enterprise groups can organise films for non-local employees, visit scenic spots and other activities, or arrange health checks for employees, which not only enriches the festival life of those supporting ‘local new year’, but also enhances employees’ sense of belonging (Han and Lin, 2021).

(2) **Focusing on the festival needs of the elderly and children in hometowns**. The concern for parents and children in their hometown is a significant concern of those who support ‘local new year’. The local government should actively collaborate with the unions and industry organisations, and rely on the street or community cadres of the registered residence of the non-locals to care for the elderly and children in their hometowns. During the Spring Festival, relatives can bless each other by means of ‘online new year greetings’ (Todd et al., 2022). For families that must be reunited, personnel mobility isolation should be adopted, such as use of special vehicles to pick up and drop people, so as to maximise the control of the spread of the pandemic.

## Conclusions and implications

Based on the public evaluation, this study used the Cp Method to analyse and extract the influencing factors of behaviour that support ‘local new year’. The primary factors that determine whether people travel home for the Chinese New Year are, ‘whether there are relatives (elderly/children) in their hometown’, ‘whether they can buy tickets’, ‘the risk of contracting infection during travel’, and ‘whether activities were arranged in city of work’. The conclusion does not support the rationality of the current cash compensation policy of ‘local new year’, indicating that people’s demand for ‘local new year’ is more spiritual than material. This study examined the relationship between the primary influencing factors by building the behaviour choice model of ‘local new year’ under the normalisation of pandemic prevention and control. Results revealed that ‘the arrangement of activities in city of work’ is the primary driving factor for the decision favouring ‘local new year’; ‘the possibility of contracting COVID-19 during travel’, and ‘whether there are relatives (elderly/children) at home’ were the secondary driving factors. According to the analysis of influencing factors and the current situation of the formulation and implementation of the ‘local new year’ policy, the implications of this study on the formulation of policies are as follows:

(1) China’s ‘local new year’ policy should be formulated region-wise in combination with the occasional and local characteristics of the current pandemic situation. According to the severity of the pandemic, the ‘local new year’ policy should be implemented with flexible systems such as ‘strict implementation’, ‘promotion and encouragement’ and ‘non coercion’ to ensure the operability and humanisation of the implementation of the policy (Collins and Welsh, 2022).

(2) China’s current ‘local new year’ policy is dominated by material compensation, which weakens the importance of people’s demand for spiritual culture and social activities. The current situation of this policy is in contradiction with the conclusion of the study, that is, the primary driving factor of activity arrangement in city of work. The existing policies cannot meet the actual interests and demands, and cannot realise the pandemic prevention policy while considering the needs of people’s spiritual culture and social activities.

(3) China’s current ‘local new year’ policy is mostly conducted for migrant workers, who are expected to return home. There is a lack of research on the children and the elderly in their hometown, and the current policy ignores the objective fact that reuniting with their relatives is an important motivation for migrant workers to return home. ‘Whether there are relatives (elderly/children) at home’ as the secondary influencing factor concerning ‘local new year’ will become the basis for policy improvement and adjustment, which is conducive to promoting the ‘local new year’ behaviour.

## Data Availability

The relevant data involved in this study are from the questionnaire survey conducted by the research team. Due to the fact that the original data involves the personal information of the interviewees, it cannot be fully disclosed at the request of some interviewees. The datasets generated during and/or analysed during the current study are available from the corresponding author on reasonable request.
